# Most Lung and Colon Cancer Susceptibility Genes Are Pair-Wise Linked in Mice, Humans and Rats

**DOI:** 10.1371/journal.pone.0014727

**Published:** 2011-02-24

**Authors:** Lei Quan, Alphons P. M. Stassen, Claudia A. L. Ruivenkamp, Tom van Wezel, Remond J. A. Fijneman, Alan Hutson, Neelima Kakarlapudi, Augustinus A. M. Hart, Peter Demant

**Affiliations:** 1 Department of Molecular and Cellular Biology, Roswell Park Cancer Institute, Buffalo, New York, United States of America; 2 Department of Genetics and Cell Biology/ Clinical Genetics, Maastricht University, Maastricht, The Netherlands; 3 Department of Human and Clinical Genetics, Leiden University Medical Center, Leiden, The Netherlands; 4 Department of Pathology, VU University Medical Center, Amsterdam, The Netherlands; 5 Department of Biostatistics, Roswell Park Cancer Institute, Buffalo, New York, United States of America; 6 Divisions of Molecular Genetics and Radiotherapy, The Netherlands Cancer Institute, Amsterdam, The Netherlands; Centro de Investigación Príncipe Felipe, Spain

## Abstract

Genetic predisposition controlled by susceptibility quantitative trait loci (QTLs) contributes to a large proportion of common cancers. Studies of genetics of cancer susceptibility, however, did not address systematically the relationship between susceptibility to cancers in different organs. We present five sets of data on genetic architecture of colon and lung cancer susceptibility in mice, humans and rats. They collectively show that the majority of genes for colon and lung cancer susceptibility are linked pair-wise and are likely identical or related. Four CcS/Dem recombinant congenic strains, each differing from strain BALB/cHeA by a different small random subset of ±12.5% of genes received from strain STS/A, suggestively show either extreme susceptibility or extreme resistance for both colon and lung tumors, which is unlikely if the two tumors were controlled by independent susceptibility genes. Indeed, susceptibility to lung cancer (*Sluc*) loci underlying the extreme susceptibility or resistance of such CcS/Dem strains, mapped in 226 (CcS-10×CcS-19)F2 mice, co-localize with susceptibility to colon cancer (*Scc*) loci. Analysis of additional *Sluc* loci that were mapped in OcB/Dem strains and *Scc* loci in CcS/Dem strains, respectively, shows their widespread pair-wise co-localization (P = 0.0036). Finally, the majority of published human and rat colon cancer susceptibility genes map to chromosomal regions homologous to mouse *Sluc* loci. 12/12 mouse *Scc* loci, 9/11 human and 5/7 rat colon cancer susceptibility loci are close to a *Sluc* locus or its homologous site, forming 21 clusters of lung and colon cancer susceptibility genes from one, two or three species. Our data shows that cancer susceptibility QTLs can have much broader biological effects than presently appreciated. It also demonstrates the power of mouse genetics to predict human susceptibility genes. Comparison of molecular mechanisms of susceptibility genes that are organ-specific and those with trans-organ effects can provide a new dimension in understanding individual cancer susceptibility.

## Introduction

Cancer is one of the leading causes of morbidity and mortality worldwide. Individual risk of sporadic cancer in populations varies greatly and is controlled by numerous low penetrance susceptibility genes [1]. Genome-wide association (GWA) studies have revealed common variants associated with risk of cancers of colon [2]–[10], lung [11]–[16], breast [17]–[23] and prostate [24]–[30], but these variants explain only a fraction of population risk [31] and their organ specificity is unknown. Lung and colon cancer are the first and second leading causes of cancer death in the United States, accounting for 28% and 9% of cancer deaths, respectively [32]. Here we report systematic tests involving three species that reveal genetic linkage and possible identity of most susceptibility genes for the two cancers.

**Figure 1:**
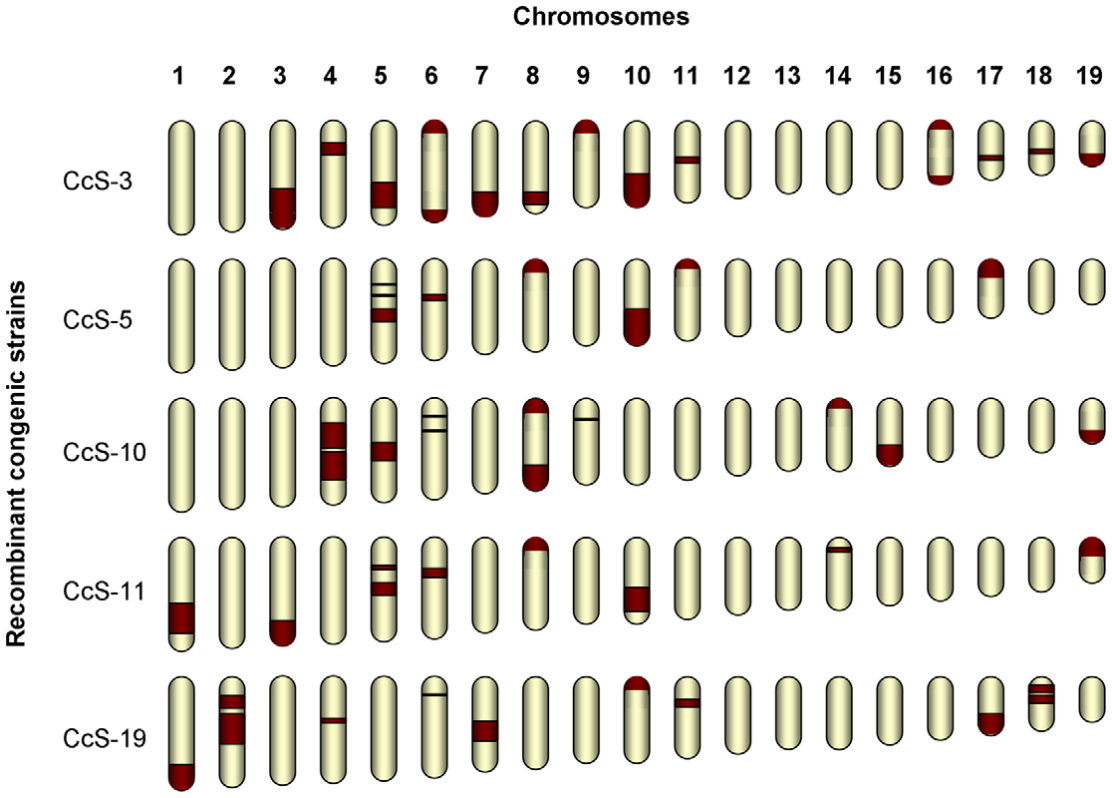
Schematic representation of the genetic composition of recombinant congenic (RC) strains. The major donor-strain regions of the CcS RC strains that were used to map colon or lung tumor susceptibility genes are shown based on real genotypes.

We analyzed mouse colon and lung cancer susceptibility genes using recombinant congenic (RC) strains, which increase the power of mapping by reducing genetic heterogeneity [33]. The RC strains were produced by two subsequent generations of backcrossing of a “donor” parental strain to a “background” parental strain, followed by twenty generations of brother-sister mating from randomly selected breeding pairs of mice. This generated a set of about 20 homozygous RC strains. Each RC strain carries a different, random set of 12.5% of “donor” strain genes and 87.5% of “background” strain genes (Figure 1) [33], [34]. In this way, the number of segregating quantitative trait loci **(**QTLs) in crosses between an RC strain and its background strain is considerably reduced and the power to detect them increased [34]. RC strains also improve QTL mapping by locating the mapped loci to relatively short donor strain-derived regions that can be precisely demarcated. Previously, we mapped 15 *Susceptibility to colon cancer* (*Scc*) loci using CcS/Dem (CcS) RC strains, derived from the ‘background’ strain BALB/cHeA (BALB/c, resistant) and ‘donor’ strain STS/A (STS, susceptible) [35]–[39]. Independently, we mapped 30 *Susceptibility to lung cancer* (*Sluc*) loci using the OcB/Dem (OcB) RC strains, derived from the ‘background’ strain O20/A (O20, susceptible) and ‘donor’ strain B10.O20/Dem (B10.O20, resistant) [40]–[42].

If a sizeable proportion of cancer susceptibility genes for the two organs are identical or genetically related, a significant number of colon and lung cancer susceptibility loci would co-localize in the same chromosomal locations. Indeed, analysis of these loci strongly indicates that most susceptibility genes for lung and colon cancer are not genetically independent but are pair-wise linked. Moreover, this co-localization is observed also between mouse lung cancer susceptibility loci and colon cancer susceptibility loci in human and rat. We show five independent sets of data including interspecies homologies, which collectively strongly suggest that most susceptibility genes for lung and colon cancer are not genetically independent as has been believed but are linked pair-wise and probably functionally related or identical.

## Materials and Methods

### Ethics Statement

All animal experiments were approved by the IACUC committee at Roswell Park Cancer Institute (permit number IACUC M905).

### Mice

Mice were maintained in ventilated filter top cages under a strict light-dark regimen and received acidified drinking water and a standard laboratory diet (LM-485, Harlan Teklad, U.S.) *ad libitum*. RC strains are inbred and form sets of about 20 strains derived from the same parental strains. Each OcB recombinant congenic (RC) strain has 87.5% of the genome from the O20 strain – the “background” strain that is relatively susceptible and 12.5% from the B10.O20 strain – the “donor” strain that is relatively resistant to lung tumors. Each CcS recombinant congenic (RC) strain has 87.5% of the genome from the BALB/c strain – the “background” strain that is relatively resistant and 12.5% from the STS strain – the “donor” strain that is relatively susceptible to colon tumors (Figure 1) [33], [34].

### Lung tumor induction and analysis in the present study

Lung tumor induction in mice has been described previously [40]. Briefly, on day 17 of gestation, the pregnant (CcS-10×CcS-19)F1 females were given an intraperitoneal (i.p.) injection of 30 mg/kg body weight of the carcinogen N-ethyl-N-nitrosourea (ENU) dissolved in phosphate-buffered citric acid (pH 5.8) [40]. The offspring of carcinogen-injected F1 females were thus exposed to ENU transplacentally. This progeny were euthanized at the age of 16 weeks and their whole lungs were removed, fixed in 10% neutral buffered formalin and embedded in histowax. For tests of lung tumor susceptibility of CcS strains, we induced lung tumors in CcS-19, CcS-11, CcS-10 and CcS-20 mice. We also induced lung tumors in crosses of CcS-19, CcS-11, CcS-10 or CcS-20 female mice with (BALB/c×FVB)F_1_ male mice (due to the small number of available CcS mice). For linkage tests, lung tumors were induced in 226 F2 intercross mice produced between CcS-10 and CcS-19 mice.

The embedded lungs were sectioned semi-serially (5-µm sections at 100-µm intervals). In most cases, we obtained 30 to 35 sections per lung. All sections were stained with haematoxylin-eosin and examined microscopically at 50X and 400X magnifications. To distinguish unequivocally individual tumors, position of a tumor in the lung lobe in sequential sections, its shape and size, positional relation to bronchi and blood vessels, and characteristics of tumor cells have been used. The tumors analyzed in this study represent a continuous histological spectrum from entirely benign adenomas (a minority) to adenocarcinomas of different degree of progression, characterized by extent of disorganization of the original organ architecture, large differences in cell morphology, pronounced nuclear pleomorphism, intra-nuclear cytoplasmic inclusions, extensive stromal areas and vascular recruitment. When allowed to develop for a longer time than in the present study, most of the tumors form advanced carcinomas with invasion of adjacent alveoli, and penetration into bronchi and blood vessels (unpublished observatins). Number of tumors, tumor size and tumor load were scored as described previously [40]. Briefly, tumor size was expressed as the sum of all measured surfaces (calculated using a grating in the ocular) in the semiserial sections where the tumor was present, and it corresponded to tumor volume. Tumors that did not exceed a diameter of 300 µm in any of the sections were not included in the data. Tumor load was calculated as the sum of the sizes (volumes) of all tumors in a mouse and it corresponded to the total tumor burden of the mouse.

### Genotyping

More than 90 % of the genetic material from the “donor” strain in a RC strain is concentrated in 9 to 13 discrete contiguous chromosomal regions with intermediate length (5–25 cM), that are usually located on 7 to 11 different chromosomes [34]. We determined the positions and length of the majority of the donor-strain derived chromosomal regions in CcS and OcB RC strains with 855 and 716 microsatellite markers across the whole genome, respectively. Based on such information, the donor strain-derived regions segregating in 226 (CcS-10×CcS19) F2 mice were PCR-genotyped [40] using 23 microsatellite markers: D1Mit291, D1Mit155, D2Mit99, D2Mit156, D2Nds3, D4Mit53, D4Mit15, D5Mit68, D6Mit177, D7Mit105, D8Mit17, D8Mit36, D9Mit254, D10Mit28, D10Mit2, D11Mit316, D14Mit11, D15Mit16, D17Mit72, D17Mit123, D18Mit17, D18Mit124, D19Mit6 (http://informatics.jax.org). Each known segregating chromosomal region is represented by at least one marker. More markers have been tested in the longer donor chromosomal regions and the maximal distance between two markers was less than 10 cM.

### Statistical analysis

#### a. Linkage and direction of allelic effects in (CcS-10xCcS-19)F_2_ Mice

The dataset of this experiment was submitted to the PLoS One website as supplemental material (Dataset S1). The chromosomal regions affecting tumor load, size and number were determined by analysis of variance (ANOVA) with the use of individual microsatellite markers listed in “genotyping” above. The effects of each marker, sex and interaction between pairs (marker-marker and marker-sex) on the corresponding phenotypes were tested by the PROC GLM (general linear models) procedure of the SAS 9.1 statistical package for Windows (SAS Institute, Inc., Cary, NC). A backward-elimination procedure was followed to exclude statistically nonsignificant effects (*P*>0.05). The P-values of the significant effects were then corrected for multiple testing using the method of Lander and Kruglyak [43] to construct the final model. All statistical tests were two-sided. Using least square (LS) means of each genotype from ANOVA we determined the number of main effects and interactions, where CcS-19-like genotypes were associated with susceptibility or resistance compared to CcS-10-like genotype (differences >30%).

#### b. Evaluation of tumor susceptibility pattern of RC strains

Colon tumor numbers and lung tumor loads or numbers were compared between the RC strains CcS-10, -11, -19, and -20 by the Wilcoxon (rank sums) two-sample tests using the PROC NPAR1WAY procedure of the SAS 9.1 statistical package for Windows (SAS Institute, Inc., Cary, NC).

#### c. Analysis of previously published Sluc and Scc loci

We used the published mapping data on the *Scc* or *Sluc* loci without any pre-selection. We identified the overlapping STS and B10.O20 donor strain-derived chromosomal regions, and determined which of the 14 *Scc* (*Scc1* and *Scc10* are considered here a single locus because they are less than 1 cM apart) and 30 *Sluc* loci map into such overlapping STS-B10.O20 donor strain-derived chromosomal regions. We used the Poisson distribution with mean parameter (mScc and mSluc), which is equal to the total number of detected loci of each type divided by the size of the total length of genome tested for that type. The probability to observe at least one locus of a particular type k in a region of size s equals 1-exp(-mk*s). The probabilities that both *Scc* and *Sluc*, none of them, or only a *Scc* or a *Sluc* locus are present are {1-exp(-mScc*s)}*{(1-exp(-mSluc*s)}, exp(-mScc*s)*exp(-mSluc*s), {1-exp(-mScc*s)}*exp(-mSluc*s), and exp(-mScc*s)*{1-exp(-mSluc*s)}, respectively, assuming the loci of the two types are distributed independently over the genome. These values were then compared with the actual data by chi-square.

#### d. Co-localization of human colon and mouse lung tumor susceptibility genes

We used published information on human colon cancer susceptibility loci detected in genome wide association or linkage studies. Orthologous regions of these loci in the mouse were compared with known *Sluc* and *Scc* loci. We evaluated by the binomial distribution test possibility of the observed number of human colon cancer susceptibility loci, whose orthologous regions were polymorphic in the tested mice, within an average 3.3cM of the published *Sluc* loci.

## Results

### Correlated susceptibility to colon and lung tumors in CcS RC strains

**Figure 2:**
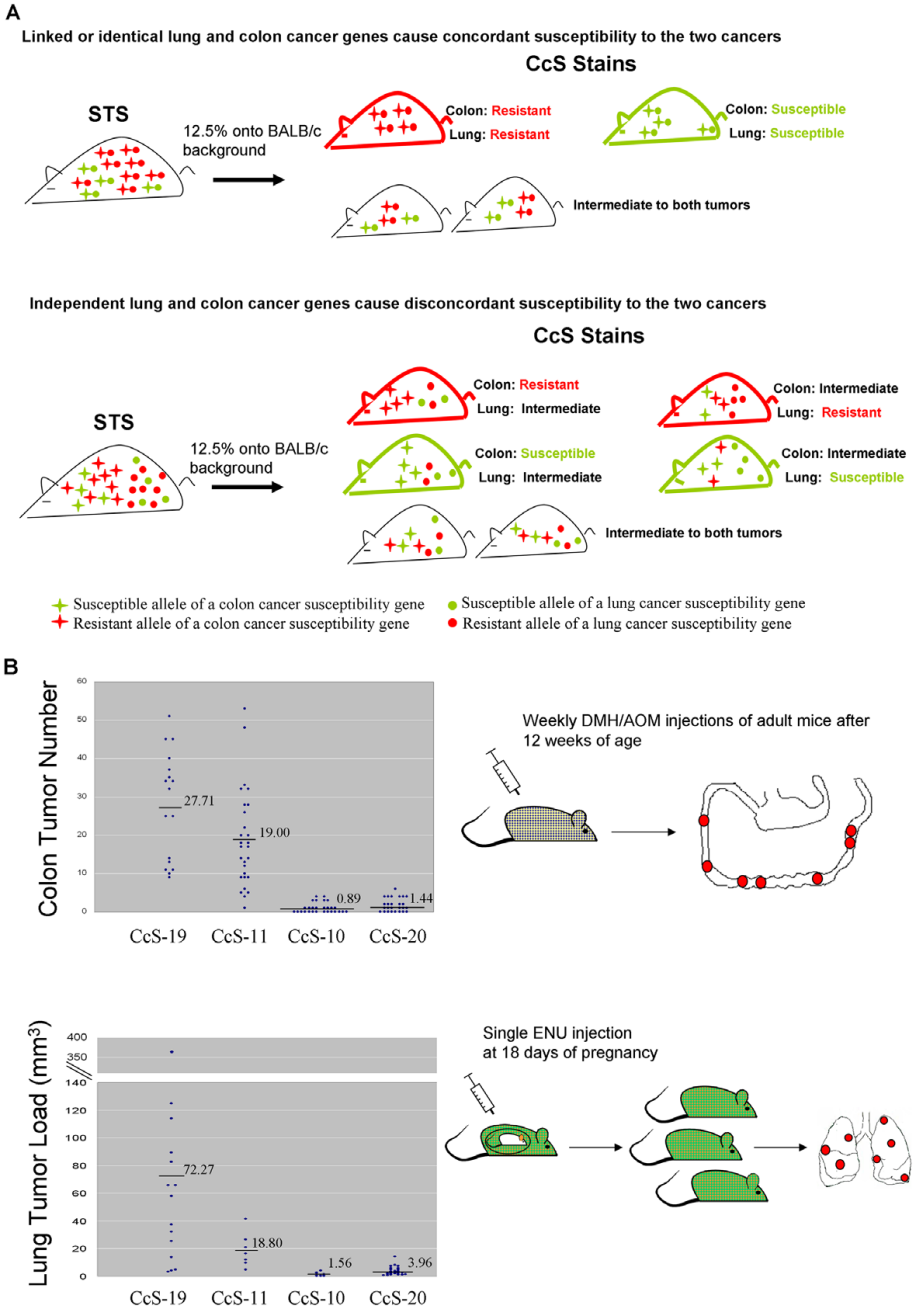
Correlated lung and colon cancer susceptibility in the CcS RC strains. **A.** Expected susceptibility to colon and lung tumors under different hypotheses. Concordant susceptibility or resistance to colon and lung tumors is expected when the majority of the susceptibility genes of the two cancers are closely linked or identical (upper panel); but not when the susceptibility genes of the two cancers are independent of each other (lower panel). **B.** Observed susceptibility to colon and lung tumors in the CcS RC strains with extreme susceptibility phenotype. Each dot represents a mouse. Mean tumor number of each strain is indicated. Upper panel: colon tumor numbers for CcS-19, CcS-11, CcS-10 and CcS-20 mice. Colon tumor number is directly proportional to colon tumor load, since in our experiments colon tumor sizes did not differ significantly among the CcS strains [35]. Lower panel: lung tumor loads for CcS-19, CcS-11, CcS-10 and CcS-20 mice. The same extreme susceptibility or resistance to lung tumors observed here has been also seen in hybrid mice between CcS and (BALB/c×FVB)F1 mice (Figure S1, Table S1).

At the first step, we selected two RC strains highly susceptible and two RC strains most resistant to colon tumors and tested their susceptibility to lung tumors. The RC strains CcS-19 and CcS-11 are highly susceptible and CcS-10 and CcS-20 are very resistant to colon tumors (p<0.0001, Figure 2B upper) [35], [37] induced by repeated injections of carcinogens 1,2-dimethyl-hydrazine (DMH) or azoxymethane (AOM). We induced lung tumors in CcS-19 and CcS-20 or CcS-11 and CcS-10 in two independent experiments. We observed that, concordant with the colon tumor susceptibility or resistance, CcS-19 is highly susceptible to ENU-induced lung tumors compared to CcS-20 (p<0.0001, Wilcoxon test), and CcS-11 is highly susceptible to ENU-induced lung tumors compared to CcS-10 (p = 0.0012, Wilcoxon test) (Figure 2B lower). The extreme susceptibility or resistance to lung tumors observed in the CcS strains, concordant to colon tumor susceptibility, has been supported by results from crosses of CcS-19, CcS-11, CcS-10 and CcS-20 with (BALB/c×FVB)F_1_ mice (tested due to the small number of available CcS mice) (Figure S1, Table S1). Mice of these crosses carry at each locus one allele of the pertinent RC strain and showed similar susceptibility pattern to the homozygous CcS mice. These data suggest that the small subsets of 12.5% STS genes received by these RC strains contain either predominantly susceptible (CcS-11, CcS-19), or predominantly resistant (CcS-10, CcS-20) alleles at most colon (*Scc*) and lung cancer (*Sluc*) genes, suggesting their pair-wise linkage or identity (Figure 2A upper). Otherwise, these RC strains would be extremely susceptible or extremely resistant to one type of tumor, but not likely to the other (Figure 2A lower).

### Scc-Sluc linkage in (CcS-10×CcS19)F2 hybrids

To elucidate the concordant extreme susceptibility of CcS-19 and resistance of CcS-10 mice to both colon and lung tumors, we mapped *Sluc* loci in ENU-treated intercross (CcS-10×CcS-19)F_2_ mice. We compared locations of these *Sluc* loci with locations of *Scc* loci previously detected in the CcS strains.

#### Mapping of Sluc loci

**Figure 3:**
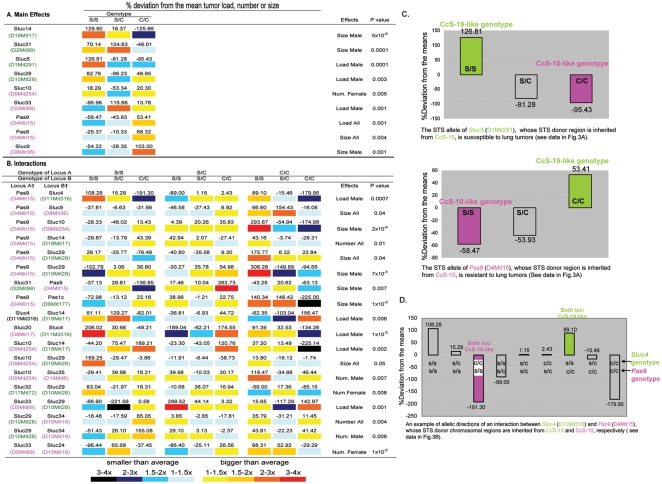
Linkage data and the estimated effects of lung cancer susceptibility (*Sluc*) loci in (CcS-10×CcS-19)F2 mice. Eight main effects (**A**) and 17 interactions (**B**) were detected. The microsatellite markers with linkage are listed below each locus. Markers in pink represent donor chromosomal region from CcS-10 and markers in green represent donor regions from CcS-19. The data are presented as percent deviations from the means of tumor load, number and size for each genotype or genotypic combination (for interactions) of the corresponding loci in female, male or both sexes (all), respectively, adjusted for the remaining markers in the model (least-squares means from the ANOVA output). The means (±SEM) of tumor number in the whole cross are: females, 4.78(±0.37); males, 4.18(±0.29); all mice, 4.48(±0.24). The means (±SEM) of tumor size per mouse (mm^3^) are: females, 2.05(±0.25); males, 2.26(±0.28); all mice 2.15(±0.19). The means (±SEM) of tumor load per mouse (mm^3^) are: females, 10.57(±1.60); males, 10.04(±1.30); all mice, 10.30(±1.03). †Loci A and B are interacting; s/s, homozygous STS; c/c homozygous BALB/c; s/c heterozygous; **C**. Examples are shown that the STS alleles of the *Sluc* loci are susceptible when they are inherited from the CcS-19 parental mice (*Sluc5*) and resistant when they are inherited from the CcS-10 parental mice (*Pas9*). **D**. Example is shown that in interactions, the genotypic combinations that are similar to the CcS-19 parental mice (CcS-19-like) are susceptible compared to the genotypic combinations that are similar to the CcS-10 parental mice (CcS-10-like). In the example, one of the interacting loci *Pas9* is inherited from CcS-10 and the other locus *Sluc4* is inherited from CcS-19.

We detected 1191 lung tumors in 226 (CcS-10×CcS-19)F_2_ mice. The 21 STS-derived regions segregating in the cross (about 23.5% of the genome) were defined in the CcS strains using 855 microsatellite markers (data not shown) and scanned in F2 hybrids with 23 microsatellite markers spaced on average 10cM apart. Mapping data of all significant linkages is in Figure 3, including p values corrected for genome-wide testing [43] and least-square means of susceptibility phenotypes of each locus. We detected 15 *Sluc* loci that affect tumor size, load and number (Figure 3A&B, Table S2). Eight of these loci had individual effects (Figure 3A) and seven loci were detected only in inter-locus interactions. We found 17 pair-wise inter-locus interactions (Figure 3B), in which the effect of one *Sluc* locus depends on the genotype of a second interacting *Sluc* locus [40], [42]. Five of these 15 *Sluc* loci are novel loci: *Sluc31* – *Sluc35,* linked to D2Mit99, D17Mit72, D5Mit68, D15Mit16 and D19Mit6, respectively (Figure 3A&B, Table S2). The other 10 *Sluc* loci are very close (0–1.5 cM, five loci) or relatively close (6–12 cM, five loci) to the positions of previously published *Sluc* or *Pas* loci (Table S2), so we could not rule out that they are duplicate detections rather than novel loci and did not assigned them novel symbols.

Effects of alleles of these loci support the hypothesis that they are responsible for the high susceptibility and resistance, respectively, of CcS-19 and CcS-10, because with few exceptions the allele obtained from CcS-19 confers a higher susceptibility than that from CcS-10 (Figure 3C). Similarly, the combination of alleles in interacting pairs of *Sluc* loci that is present in CcS-19 is more susceptible than that present in CcS-10 (Figure 3D).

#### Scc-Sluc co-localization

**Figure 4:**
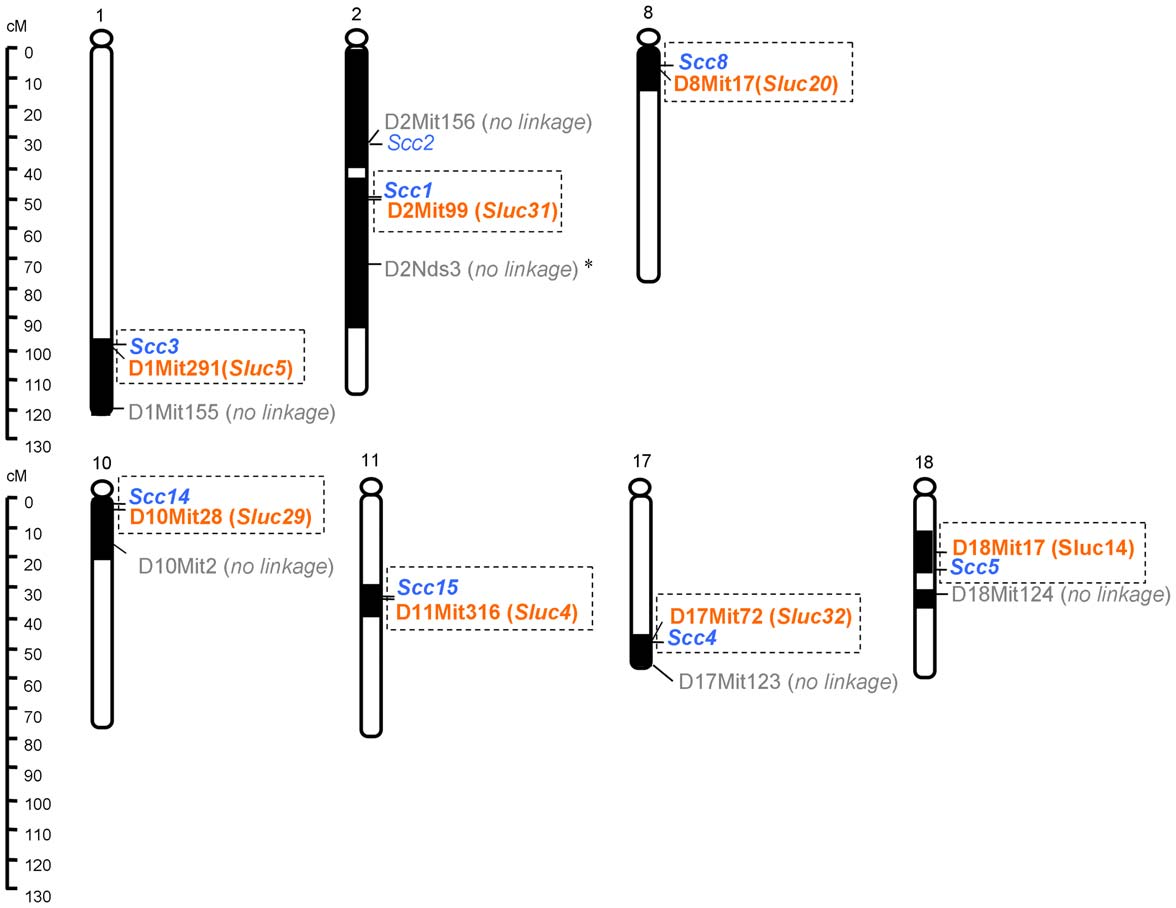
*Sluc* loci detected in (CcS-10×CcS-19)F_2_ mice co-localize with previously detected *Scc* loci. The seven *Sluc* loci in the chromosomal regions that have been tested for colon cancer susceptibility previously are shown. All the seven *Sluc* loci co-localize with *Scc* loci. Markers with linkage are highlighted in orange and the corresponding *Sluc* loci are listed. Additional markers tested on the same donor chromosomal regions that did not show linkage can help to limit the candidate regions and are shown in grey color. Previously detected *Scc* loci are highlighted in blue. Detailed information of each locus is listed in Suppl. Table 2 and Suppl. Table 3.

We compared the map location of the 15 *Sluc* loci detected in the (CcS-10×CcS-19)F_2_ hybrids with location of *Scc* loci detected previously in crosses of CcS strains -3, -5, -11, -19, with BALB/c (Table S3)[36], [38], [39]. Seven of the 15 *Sluc* loci, linked to D1Mit291, D2Mit99, D8Mit17, D10Mit28, D11Mit316, D17Mit72 and D18Mit17, mapped to regions that had been previously tested for colon cancer susceptibility. Without exception, they all co-localized with *Scc* loci and formed linked pairs of *Scc3*/*Sluc5*, *Scc1*/*Sluc31, Scc8*/*Sluc20*, *Scc14*/*Sluc29*, *Scc15*/*Sluc4, Scc4*/*Sluc32* and *Scc5*/*Sluc14* (Figure 4, Table S2). Five of these *Sluc* loci mapped less than 1cM from the paired *Scc* locus; one locus 2 cM and one 5 cM. The other eight newly detected *Sluc* loci are located in regions that were not yet tested for colon tumor susceptibility in RC strains and could pair with presently unknown *Scc* loci. These data show that the *Scc* and *Sluc* loci underlie the concordant extreme susceptibility or resistance to colon and lung tumors and are pair-wise clustered.

### Scc-Sluc linkage in independent strains

**Figure 5:**
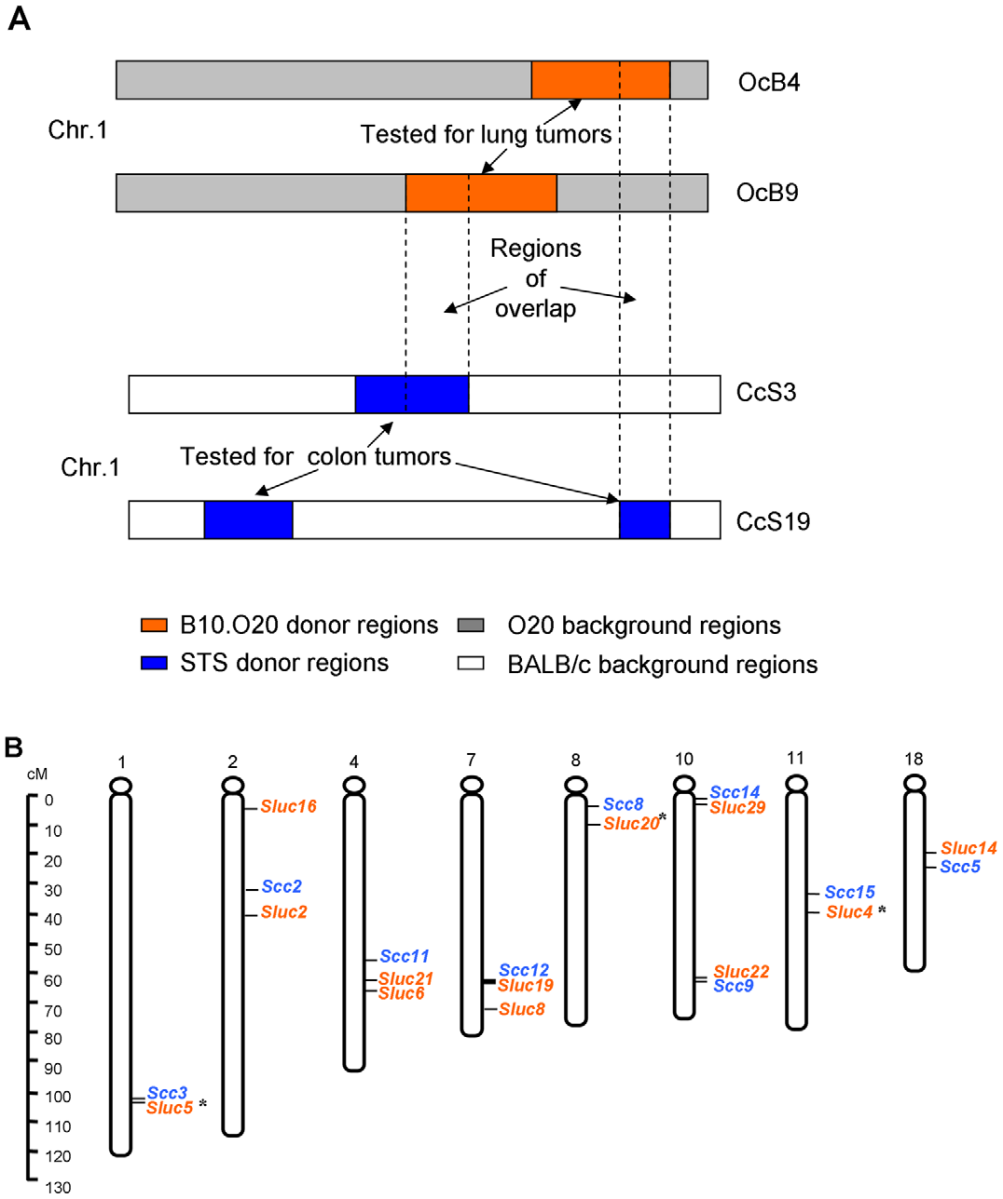
Co-localization between *Sluc* loci mapped in the OcB RC strains and *Scc* loci mapped in the CcS RC strains. **A**. Schematic representation of overlapping donor chromosomal regions between the CcS and OcB RC strains (regions tested for both colon and lung cancer susceptibility loci). Such regions are informative and we used them to test whether *Sluc* and *Scc* loci are more frequently located together in the same donor chromosomal region. Part of a chromosome is shown as example. **B**. Frequent co-localization between *Sluc* and *Scc* loci identified independently in OcB and CcS RC strains, respectively (See also Table 1 for detailed locations). *Map locations of these *Sluc* loci are slightly different from the locations of the same loci shown in Figure 4, since they are mapped in different RC strains using different microsatellite markers.

We performed an analysis of map locations of *Scc* loci and *Sluc* loci that were previously mapped in two completely independent projects using crosses of four CcS strains to map *Scc* genes [36], [38], [39] and five OcB strains to map *Sluc* genes [40]–[42], respectively (Table S3). The donor strain chromosomal regions of the CcS strains tested for colon tumor susceptibility comprised together about 40–50% of the genome, which is similar to the total proportion of the genome of the OcB strains tested for lung tumor susceptibility. The ‘overlap’ regions screened for both lung and colon tumor susceptibility can be used to evaluate the possible co-localization of *Scc* and *Sluc* genes, whereas the ‘non-overlap’ regions were studied for only one tumor type and hence are not informative (Figure 5A). We identified 23 ‘overlap’ chromosomal regions with a total length of 430 cM (‘overlap’ in Table 1), containing 9 *Scc* loci and 14 *Sluc* loci (Table 1). We found that the “concordant ‘overlap’ regions” containing either both a *Scc* and a *Sluc* locus (n = 9) or none of them (n = 11) outnumber vastly the “discordant ‘overlap’ regions” that contain either only a *Scc* (n = 0) or only a *Sluc* locus (n = 3) (Table 1). These observed frequencies are significantly different from the expected frequencies assuming independent distribution of *Scc*-*Sluc* loci, as calculated from Poisson distribution based on length of the overlap regions (P = 0.0036, modified 2×2 test–Materials and Methods). These data indicate that the genetic relatedness between a large number of colon and lung cancer susceptibility genes that we observed in the CcS RC strains is likely common in other mouse strains as well. In fact, the p value 0.0036 likely underestimates the actual significance of the co-localization, because in the nine overlap regions that contain both a *Scc* locus and a *Sluc* locus, the distances between the markers for these loci are about 75% shorter than the length of the overlap regions (derived from Table 1, Figure 5B).

**Table 1:** *Sluc* and *Scc* loci identified independently in OcB and CcS strains frequently co-localize in the same donor chromosomal region.

Chr	Colon Tumors – tested in CcS strains			Overlap*	Lung Tumors – tested in OcB strains		
	*Scc* locus (cM)	Tested Regions (cM)	Regions with Linkage (cM)		Regions with Linkage (cM)	Tested Regions (cM)	*Sluc* locus (cM)
**1**	none	32.8–41		32.8–41		0–59	none
**1**	***Scc3*** (101.5)	81.6–127	100–127	**81.6**–**127**	81.6–127	81.6–127	***Sluc5*** (100†)
**2**	***Scc2*** (32)	5–41.4	5–41.4	**5**–**41.4**	5–42.7	0–47.5	***Sluc2*** (41)
**2**					0–10	0–47.5	*Sluc16* (5)
**2**	none	45–95.5		69–95.5		69–114	none
**3**	none	6.7–11.2		6.7–11.2		0–45.2	none
**4**	none	20.8–40		20.8–40		0–81	none
**4**	***Scc11*** (57.4)	56.5–62.3	56.5–62.3	**56.6**–**62.3**	0–62.3	0–81	***Sluc21*** (62.3)
**4**					56.6–81	0–81	***Sluc6*** (67)
**5**	none	61–78		64–78		64–92	none
**6**	none	0–20.5		2.8–20.5	2.8–16	2.8–26.5	*Sluc7* (6)
**6**	none	42–61.4		42–48.7		36.5–48.7	none
**6**	none			58.6–61.4	58.6–63.6	58.6–63.6	*Sluc3 (61.2)*
**6**	none	62.5–75		67–75		67–75	none
**7**	none	8–74		8–15		0–15	none
**7**	***Scc12*** (63.5)	8–74	60–74	**28.7**–**74**	51.8–66	28.7–74	***Sluc19*** (63.5)
**7**					28.7–74	28.7–74	***Sluc8*** (72.0)
**8**	***Scc8*** (4)	0–19.5	0–16	**0**–**19.5**	0–31.5	0–31.5	***Sluc20*** (10)
**8**	none	41–67		53–67	53–73	53–73	*Sluc9* (59)
**10**	***Scc14*** (2)	0–21	0–21	**0**–**21**	2–36	0–36	***Sluc29*** (4)
**10**	***Scc9*** (63)	49–77	62–77	**51.5**–**77**	56–77	51.5–77	***Sluc22*** (61)
**11**	***Scc15*** (33.9)	20–40	30–40	**27.9**–**40**	27.9–40	27.9–40	***Sluc4*** (40)
**16**	none	0–28.2		0–27.6		0–27.6	none
**18**	***Scc5*** (25)	2–26	5–26	**2**–**26**	0–24	0–41	***Sluc14*** (20)
**18**	none	31–37		31–37		31–37	none
**19**	none	4.5–41		4.5–41		4.5–53	none

*The ‘overlap’ chromosomal regions that have been tested for both colon cancer susceptibility in CcS RC strains and lung cancer susceptibility in OcB RC strains are listed. The regions containing a *Scc* gene as well as a *Sluc* gene are highlighted in bold.

† Lung tumor susceptibility *Sluc5* has been mapped in separate experiments at 87 and 100 cM, respectively (Tripodis et al. 2001); the position at 100 cM is used for the analysis.

Several laboratories carried out productive searches for colon [44]–[46] and lung [47]–[52] cancer susceptibility genes. However, we could not include these published cancer susceptibility loci into the present analyses because the candidate regions of most of them cannot be defined as precisely as the donor-strain regions in RC strains, so the extent of their overlaps cannot be evaluated statistically. Also, the detection of co-localization depends on the power of the mapping test, which is less in whole genome crosses than in RC crosses [34]. Nevertheless, these data showed that the colon cancer susceptibility locus *Ccs1* detected in ICR×C57Bl/6 backcross [44], maps only 1cM from the lung cancer resistence locus *Par3* detected in SMXA×A backcrosses [50] on mouse chromosome 12.

### Mouse Sluc loci co-localize with mouse orthologues of human and rat colon cancer susceptibility loci

**Figure 6:**
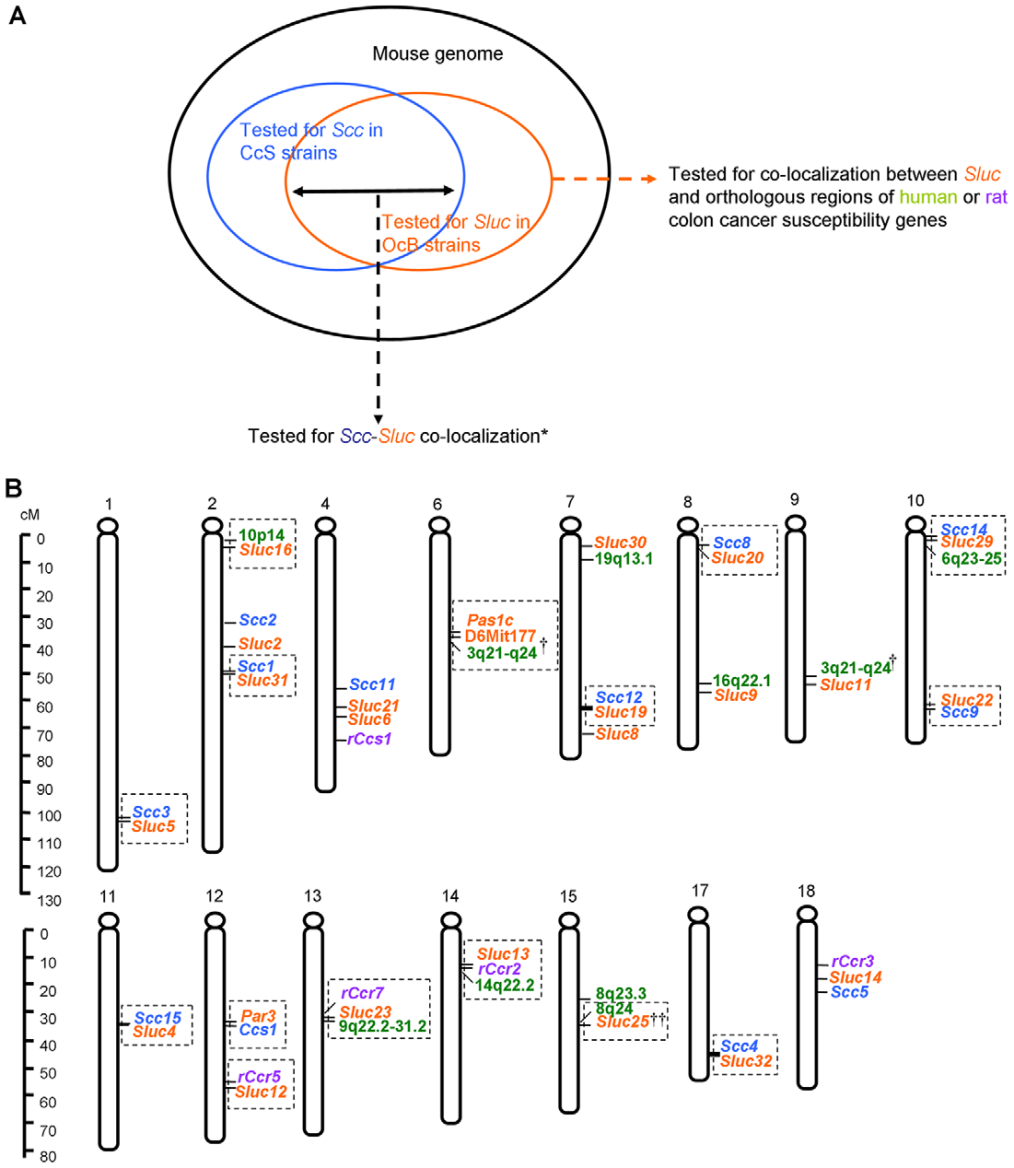
Interspecies correlation between colon and lung cancer susceptibility loci. **A.** Schematic representation of the part of genome used for the co-localization analyses. The *Sluc* loci analyzed here included also 2 *Sluc* loci identified in (CcS10 XCcS19)F2 mice and 1 *CcS* locus [44] and 1 *Par* locus [50] identified by other group. **B.** Interspecies correlation between colon and lung cancer susceptibility loci. This figure summarizes all 21 clusters of colon and lung cancer susceptibility loci mapped in mouse RC strains (orange for lung, blue for colon), human colon (green) and rat colon (purple). Clusters in which the lung and colon cancer loci mapped within 2.5cM of each other are highlighted in squares. Most colon and lung cancer susceptibility loci co-localize, with the exception of human 15q13 and 20p12.3 (colon), and rat *rCcr6* and *rCcr8* (colon). Orthologous regions of human 18q21, 11q23 (colon) and 5p15, 6p21 and 15q25 (lung), and rat *rCcr1*, *rCcr4* and *rCcr9* (colon) are not informative since they were not tested for lung or colon cancer susceptibility in mouse RC strains. †*Pas1c* has also been detected in our (CcS-10×CcS-19)F2 cross at D6Mit177. Human colon cancer locus 3q21-q24 is mapped to an 18Mb region and orthologous to two mouse chromosomal regions: Chr.6 (*Pas1c*) and Chr.9 (*Sluc11*), respectively. †† Two human colon cancer susceptibility loci co-localize with a *Sluc* locus.

Co-localization of colon and lung cancer susceptibility genes in mouse suggests that many of them may be related or identical. We therefore investigated possible parallels of this finding in humans and rats (Figure 6A).

#### Human colon cancer susceptibility loci

Genome-wide association and linkage studies in humans detected 13 susceptibility loci for colorectal cancer and four for lung cancer [2]–[10]. The orthologous regions of 11 out of 13 human colon cancer susceptibility loci are polymorphic in the OcB or CcS strains that were tested for lung tumor susceptibility. Surprisingly, nine of these 11 regions are close to previously detected *Sluc* loci (Figure 6B). Six are very close (0–2.5 cM): 8q24/*Sluc25*, 9q22.2–31.2/*Sluc23*, 10p14/*Sluc16*, 3q21–24/ *Pas1c*-D6mit177 (or *Sluc11*), 14q22.2/*Sluc13* and 6q23–25(both colon and lung)/*Scc14/ Sluc29* [2], [3], [5], [6], [8], [10], [11], [39], [42], [51]; Three are relatively close(5–7cM): 8q23.3/*Sluc25*, 16q22.1/*Sluc9* and 19q13.1/*Sluc30* [8], [10], [42]. No *Sluc* locus was detected near orthologues of 15q13 or 20p12.3 [7], [8]. The fact that nine of the 11 human colon cancer susceptibility loci, whose orthologues have been tested in mouse RC strains, map on average to 3.3 cM from a *Sluc* locus, suggests that lung cancer and colon cancer susceptibility loci are also significantly clustered in humans (*P* = 0.0015, binomial distribution test). Orthologues of the colon cancer susceptibility loci at 18q21 [4] and 11q23 [9] and the lung cancer susceptibility loci at 5p15, 6p21 and 15q25 [12]–[14], are in regions that were not tested for *Sluc* or *Scc* loci.

#### Rat colon cancer susceptibility loci

Ten rat colon cancer susceptibility loci were reported [53]. The orthologous regions of seven of them are polymorphic in OcB or CcS strains tested for lung cancer susceptibility. Five of these seven regions co-localize with mouse lung cancer susceptibility loci within a distance of 2–8 cM, forming pairs of *rCcr2*/*Sluc13*, *rCcr3*/*Sluc14*, *rCcr5*/*Sluc12*, *rCcr7*/*Sluc23* and *rCcs1*/*Sluc6* (Figure 6B). No *Sluc* locus was detected in the region orthologous to rat *rCcr6* or *rCcr8*. The orthologues of *rCcr1*, *rCcr4* and *rCcr9* are in regions not tested for *Sluc* or *Scc* loci.

The orthologous regions of most human and rat colon cancer susceptibility loci are not polymorphic in the mouse strains that were tested for colon cancer susceptibility. Therefore their co-localization with *Scc* loci could not be assessed.

## Discussion

### Colon and lung cancer susceptibility genes are related or identical

The five independent sets of presented data contradict the notion of independent genetic control of colon and lung tumor susceptibility and suggest that the two classes of susceptibility genes are functionally or genetically related, or identical: **i.** Concordant high susceptibility or high resistance to both tumors in several CcS strains suggests linkage of *Scc* and *Sluc* genes received from STS (Figure 2, Figure S1, Table S1). **ii.** Indeed, in F2 hybrids between highly susceptible CcS-19 mice and highly resistant CcS-10 mice, each segregating *Scc* locus is linked with a *Sluc* locus (Figure 4). **iii.** Most *Scc* loci that were detected in crosses of CcS strains are pairwise linked with the *Sluc* loci that were detected in crosses of OcB strains (Figure 5). **iv–v.** Most mouse orthologues of human and rat colon cancer susceptibility loci co-localize with the mouse *Sluc* loci (Figure 6). Overall, 12/12 mouse *Scc* loci, 9/11 human and 5/7 rat colon cancer susceptibility loci are close to a *Sluc* locus or its homologous site, forming 21 clusters of lung and colon cancer susceptibility loci from one, two or three species. Importantly, lung and colon cancer susceptibility loci from 15 of these clusters mapped within 2.5 cM of each other (Figure 6). This evidence is hardly compatible with genetic independence of most of colon and lung tumor susceptibility loci.

Multi-organ specificity of cancer susceptibility genes is also supported by epidemiological studies in humans that revealed familial aggregations of different types of cancers, which do not correspond to known cancer syndromes [54], [55]. Although no aggregations of colon and lung cancer were found, this could be due to the distinct environmental etiology of lung cancer in humans, or because aggregation between tumors of other organs is even stronger. We also compared the map locations of *Sluc*/*Scc* loci with the location of 16 published skin cancer susceptibility (*Skts*) loci [56]–[60]. It seems that the genetic control of skin cancer might also be related to that of lung and colon, but to a much lesser extent (data not shown).

### Possible mechanisms underlying the co-localizing colon and lung cancer susceptibility genes

Molecular interpretation of our data is limited because the candidate genes for most colon and lung cancer susceptibility loci are not known. A co-localization alone need not necessarily indicate relatedness or identity. However, we have demonstrated statistically significant pair-wise co-localization of a large majority of colon and lung cancer susceptibility loci, which is incompatible with their complete or extensive independence. The nature of the relatedness need not be the same for each *Scc*-*Sluc* locus. In some *Scc*-*Sluc* loci a single gene can affect susceptibility to both tumors. The genes that affect common cell autonomous pathways might affect susceptibility to several types of cancer [61]. The co-localizing *Sluc4*/*Scc15* are linked to *Trp53*; *Sluc32*/*Scc4* are linked to mismatch repair genes *Msh2* and *Msh6*; and *Sluc30* is linked to base pair repair gene *Xrcc1*. These genes are associated with both colon and lung cancer risk [62]–[66]. However, such linkage should be interpreted with caution. For example, lung cancer susceptibility locus *Pas1* is linked to *Kras*, but other genes in the region are more likely candidates [67], [68]. As both colon and lung derive from primordial gut, cellular regulatory pathways in the two organs may partly involve the same genes. Systemic influences on tumorigenesis might also affect several tumor types, as might be the case for genes that modify such systemic reactions. Two *Scc/Sluc* pairs co- localize with functional polymorphisms of the immune system that may affect host-tumor interactions: *Scc8/Sluc20* are linked to *Marif2*, *Cinda5*, and *Lynf4*, which control macrophage and lymphocyte activation and lymphocyte infiltration of tumors, respectively, and *Scc15/Sluc4* are linked to *Cinda1* [69]–[71]. Four *Scc/Sluc* pairs, *Scc3*/*Sluc5*, *Scc1*/*Sluc31*, *Scc11*/*Sluc21* and *Scc15*/*Sluc4*, co-localize with microRNA genes [72], some of which could play an essential role in tumorigenesis [73]. In other *Scc*-*Sluc* pairs the two loci may represent duplicated genes whose function diverged into regulating tumorigenesis in the two organs. Still others may contain linked regulatory elements with tissue specific effects, such as the human 8q24 gene desert region that modifies susceptibility to five tumor types [74]. Some instances of *Scc*-*Sluc* clustering may reflect the phenomenon of non-random distribution of genes and the presence of clusters of functionally related or co-regulated genes in the genome [75], including functionally related QTLs [76]–[78]. Finally, susceptibility QTLs upon close analysis may turn out to be complex. Therefore the co-localizing colon and lung cancer susceptibility loci described here may be consisting of multiple closely linked genes, with some overlap between the two sets. The dual specificity of *Scc*-*Sluc* loci is unlikely due to carcinogen processing, as the two tumors were induced by different carcinogens acting in different ways and at different differentiation stages. DMH and AOM are metabolically activated in liver and primarily excreted into the intestine through bile, where they act locally to mutagenize intestinal epithelial cells causing G→A transitions [79]. On the other hand, ENU acts by directly modifying DNA without any prior enzymatic activation causing a wider spectrum of alterations [80], [81]. Prenatal treatment of mice with ENU at day 18 of pregnancy causes predominantly lung tumors but no colon tumors. Moreover, relative strain susceptibility to lung tumors is largely independent on the carcinogen used [82], and susceptibility loci for urethane-induced lung tumors also co-localize with *Sluc* and *Scc* loci [34] (Figure 3).

### Mouse genetic mapping predicts chromosomal location of human cancer susceptibility genes

GWA studies of cancer susceptibility [83] confirmed the genetic basis of common cancer [84] and enabled uncovering novel pathways of tumorigenesis [85], [86]. However, GWA data cannot yet identify high risk individuals, explain familial cancer clusters [87], nor identify the responsible genes, so the loci cannot be experimentally validated [31]. It has been shown previously that individual QTLs identified in rodent cancer susceptibility studies may provide a strong guide to identification of cancer susceptibility genes in humans [59], [88], [89]. The present data extend this potential by showing that the orthologous regions of most susceptibility genes for one class of cancer (lung cancer) identified in mice could systematically predict susceptibility genes for another class of cancer (colon caner) in human. This potential contribution is enhanced by several characteristics of mouse crosses: **i.** detection of polymorphic susceptibility alleles is independent on gene frequency of their human homologues and hence can reveal rare alleles in humans; **ii.** the power of mouse RC crosses is very high (1 locus per 29 tested F2 mice [34]) resulting in detection of large numbers of loci; **iii.** gene-gene interactions can be readily detected [37], [40], [42], [90]; **iv.** susceptibility genes can be molecularly identified [59], [67], [68], [91] and **v.** a hypothesis free ‘candidate region’ approach can improve detection power in human studies by strongly diminishing the multiple testing penalties.

In conclusion, this is to our knowledge the first systematic study of organ specificity of cancer susceptibility. It indicates that many lung and colon cancer susceptibility genes are linked and possibly identical. Consequently, the presently prevailing organocentric approach to cancer susceptibility may be enriched by comparing systematically the organ-specific pathways with those active in several organs. Application of this finding may also enhance effectiveness of GWA studies of cancer susceptibility in humans.

## Supporting Information

Table S1 Supplementary Table 1.
(0.03 MB DOC)

Table S2 Supplementary Table 2.
(0.05 MB DOC)

Table S3 Supplementary Table 3.
(0.10 MB DOC)

Figure S1 Supplementary Figure 1.
(0.28 MB TIF)

Dataset S1 The mapping spreadsheet for linkage analysis.
(0.10 MB XLS)
