# Supplementary material for: Most Lung and Colon Cancer Susceptibility Genes Are Pair-Wise Linked in Mice, Humans and Rats
Source: PLoS One. 2011 Feb 24;6(2):e14727. doi: 10.1371/journal.pone.0014727 (PMC3044722; doi:10.1371/journal.pone.0014727)
Supplement: Table S1 — Supplementary Table 1. (0.03 MB DOC) [file pone.0014727.s001.doc]

**Table S1. Concordant lung and colon cancer susceptibility in CcS strains**

| Strain | Comparison* | Colon tumor number p-value† | Lung tumor number p-value†† |
| --- | --- | --- | --- |
| CcS-19 | > CcS-10 | **<0.0001** | **0.0039** |
| CcS-19 | > CcS-20 | **<0.0001** | **0.018** |
| CcS-11 | > CcS-10 | **<0.0001** | **0.0061** |
| CcS-11 | > CcS-20 | **<0.0001** | **0.017** |
| CcS-19 | ≈ CcS-11 | 0.05 | 0.59 |
| CcS-10 | ≈ CcS-20 | 0.15 | 0.89 |

* The > sign indicates ‘much more susceptible than’; and the ≈ sign indicates similar susceptibility. †Based on data presented in Figure 2B. †† Based on data presented in Suppl. Figure 1.
