# Supplementary material for: Most Lung and Colon Cancer Susceptibility Genes Are Pair-Wise Linked in Mice, Humans and Rats
Source: PLoS One. 2011 Feb 24;6(2):e14727. doi: 10.1371/journal.pone.0014727 (PMC3044722; doi:10.1371/journal.pone.0014727)
Supplement: Table S2 — Supplementary Table 2. (0.05 MB DOC) [file pone.0014727.s002.doc]

**Table S2. Summary of *Sluc* loci detected in the (CcS-10 x CcS-19)F2 mice.**

| **Maker** | **Chr.** | **Position**  **(cM)** | **Maximal Regions (cM)** | ***Scc* Loci*** | *Sluc* and *Pas* Loci* | **Donor**  **Origin**† |
| --- | --- | --- | --- | --- | --- | --- |
| **D1Mit291‡** | 1 | 101.5 | D1Mit403(99.5)-D1Mit155(112.0) | *Scc3* [37] | *Sluc5* [41] | **CcS19** |
| **D8Mit17** | 8 | 4 | D8Mit3(4.0)-D8Mit125(19.2) | *Scc8* [38] | *Sluc20* [42] | **CcS10** |
| **D10Mit28‡** | 10 | 4 | Acromere(0)-D10Mit2(16) | *Scc14* [39] | *Sluc29* [42] | **CcS19** |
| **D11Mit316** | 11 | 33.7 | D11Mit26(33.9)-Trp53(39.0) | *Scc15* [39] | *Sluc4* [40] | **CcS19** |
| **D18Mit17‡** | 18 | 20 | D18Mit83(11.4)-D18Mit124(32.0) | *Scc5* [37] | *Sluc14* [41] | **CcS19** |
|  |  |  |  |  |  |  |
| **D2Mit99‡** | 2 | 50.3 | D2Mit37(44.1)-D2Nds3(73.0) | *Scc1* [36] | ***Sluc31*** | **CcS19** |
| **D17Mit72‡** | 17 | 47.4 | D17Mit38(45.3)-D17Mit123(56.7) | *Scc4* [37] | ***Sluc32*** | **CcS19** |
| **D5Mit68** | 5 | 65 | D5Mit10(54.0)-D5Mit159(67.0) | Not Tested | ***Sluc33*** | **CcS10** |
| **D15Mit16** | 15 | 61.7 | D15Mit41(54.5)-D15Mit40(65.0) | Not Tested | ***Sluc34*** | **CcS10** |
| **D19Mit6** | 19 | 55 | D19Mit35(53)-D19Mit137(55.70) | Not Tested | ***Sluc35*** | **CcS10** |
|  |  |  |  |  |  |  |
| **D6Mit177** | 6 | 38.5 | D6Mit284(37)-D6Mit165(42) | Not Tested | *Pas1c* [51] | **CcS10** |
| **D4Mit15 ‡§** | 4 | 42.6 | D4Mit53(26.6)-D4Mit11(57.4) | Not Tested | *Pas9* [51], *Sluc21* [42] | **CcS10** |
| **D8Mit36** | 8 | 67 | D8Mit14(67.0)-D8Mit92(71.4) | Not Tested | *Sluc9* [41] | **CcS10** |
| **D9Mit254** | 9 | 25 | D9Mit253(21.0)-D9Mit154(27.0) | Not Tested | *Sluc10* [41] | **CcS10** |
| **D14Mit11** | 14 | 0.7 | Acromere(0)-D14Mit44(10.0) | Not Tested | *Sluc13* [41] | **CcS10** |

*The co-localizing *Scc*, *Sluc* or *Pas* loci that were published previously are listed with references. Five loci detected in this study are new and designated as *Sluc31*-*Sluc35* (highlighted in bold).

†The linkages were detected in STS donor chromosomal regions inherited from either CcS-10 or Ccs-19 mice.

**‡**Extra markers in the neighboring regions have been tested, respectively, showing no or reduced linkage and therefore delimit the candidate region: D1Mit155 (112cM), D10Mit2 (16cM), D18Mit124 (32cM), D2Mit156(32cM), D2Nds3(73cM), D17Mit123(56.7cM), D4Mit53(26.6cM).

**§**The previously reported *Scc11* can not be related to this linkage, since both CcS-10 and CcS-19 carry the STS-derived donor fragment in the *Scc11* region.
