# Supplementary material for: Most Lung and Colon Cancer Susceptibility Genes Are Pair-Wise Linked in Mice, Humans and Rats
Source: PLoS One. 2011 Feb 24;6(2):e14727. doi: 10.1371/journal.pone.0014727 (PMC3044722; doi:10.1371/journal.pone.0014727)
Supplement: Table S3 — Supplementary Table 3. (0.10 MB DOC) [file pone.0014727.s003.doc]

**Table S3. Detailed information of previously mapped *Sluc* and *Scc* loci.**

| **Loci** | **Marker** | **Chr.** | **Pos (cM)** | **Cross** | **Maximal Region (cM)** |
| --- | --- | --- | --- | --- | --- |
| *Sluc1* | D19M9 | 19 | 47 | OcB-9,3,6 | D19mit63(24)-D19M26(51) |
| *Sluc2* | D2M56 | 2 | 41 | OcB-9,4 | D2mit367(26.2)-D2mit35(45) |
| *Sluc3* | D6M218 | 6 | 61.2 | OcB-9 | D6mit334(60.75)-D6mit13(63.6) |
| *Sluc4* | D11M15 | 11 | 40 | OcB-9 | D11Nds9(29.2)-D11Nds1(43.8) |
| *Sluc5* | D1M36 | 1 | 92.3 | OcB-4 | D1mit33(81.6)-D1mit456(95.8) |
| *Sluc5* | D1M221 | 1 | 102 | OcB-16,6 | D1mit403(100)-Telemere(112) |
| *Sluc6* | D4M158 | 4 | 67 | OcB-9 | D4mit37(56.5)-D4mit42(81) |
| *Sluc7* | D6M158 | 6 | 6 | OcB-4 | D6mit1(2.8)-D6mit184(26.35) |
| *Sluc8* | D7NDS4 | 7 | 72.4 | OcB-9 | D7mit66(57.5)-end (74) |
| *Sluc9* | D8M35 | 8 | 59 | OcB-4 | D8mit47(53)-D8mit56(73) |
| *Sluc10* | D9M2 | 9 | 17 | OcB-4 | D9mit138(8)-D9mit45(24) |
| *Sluc11* | D9M12 | 9 | 55 | OcB-4 | D9mit32(35)-D9mit15(61) |
| *Sluc12* | D12Nds2 | 12 | 59 | OcB-16 | D12mit6(45)-telomere(61) |
| *Sluc12* | D12Nds2 | 12 | 59 | OcB-16,6 | D12mit27(52)-D12mit144(61) |
| *Sluc13* | D14M120 | 14 | 12.5 | OcB-6 | D14mit126(5.0)- D14mit37(27.5) |
| *Sluc14* | D18M17 | 18 | 20 | OcB-4 | Acromere(0)-D18mit56(23) |
| *Sluc15* | D1M170 | 1 | 19.5 | OCB3 | Acromere(0)-D1mit5(32.8) |
| *Sluc16* | D2M5 | 2 | 5 | OcB-6,4 | Acromere(0)-D2mit465(10) |
| *Sluc17* | D2M200 | 2 | 107 | OcB-16 | D2mit148(105)-Telemere(110) |
| *Sluc18* | D4M4 | 4 | 12.1 | OcB-6,4 | D4mit41(10.5)-D4mit28 (48.5) |
| *Sluc19* | D7M105 | 7 | 63.5 | OcB-16 | D7mit53(51.8)- D7mit12(66) |
| *Sluc20* | D8M3 | 8 | 10 | OcB-9,16 | Acromere(0)-D8mit65(22.5) |
| *Sluc21* | D4M70 | 4 | 62.3 | OcB-16 | D4mit164(28.6)-D4Mit134(62.3) |
| *Sluc22* | D10M122 | 10 | 61 | OcB-16 | D10mit12(56)-Telomere (70) |
| *Sluc23* | D13M139 | 13 | 32 | OcB-3 | D13mit165(30)-D13mit13(35) |
| *Sluc24* | D15M13 | 15 | 6.7 | OcB-16 | Acromere(0)- D15mit6(13.7) |
| *Sluc25* | D15Nds3 | 15 | 32 | OcB-4 | D15mit22(15.4)-D15mit171(54.5) |
| *Sluc26* | D15M96 | 15 | 48.9 | OcB-4,16 | D15mitNds3(32)-D15mit171(54.5) |
| *Sluc27* | D16M19 | 16 | 54 | OcB-16 | D16mit67(47.0)-D16mit191(57.8) |
| *Sluc28* | D18M7 | 18 | 50 | OcB-16 | D18mit49(49.0)-Telemere(60) |
| *Sluc29* | D10M28 | 10 | 4 | OcB-4 | D10mit49(2)-D10mit31(36) |
| *Sluc30* | D7M57 | 7 | 4 | OcB-3 | acromere(0)-D7mit55(15) |
| *Scc1* | D2M14 | 2 | 49.6 | CcS-19 | *Ptprj* |
| *Scc2* | D2M156 | 2 | 32 | CcS-19 | D2M5(5)-D2M92(41.4) |
| *Scc3* | D1M208 | 1 | 101.5 | CcS-19 | D1M403(100)-Telomere(127) |
| *Scc4* | D17M72 | 17 | 47.4 | CcS-19 | D17M38(45.3)-D17Nds4(Syn) |
| *Scc5* | D18M57 | 18 | 25 | CcS-19 | D18M149(24)-D18M54(26) |
| *Scc6* | D11M2 | 11 | 2.4 | CcS-5 | Acromere(0)-D11M162(8) |
| *Scc7* | D3M163 | 3 | 87.6 | CcS-3 | D3M113(72.9)-telomere(87.6) |
| *Scc8* | D8M17 | 8 | 4 | CcS-3 | Acromere(0)-D8M64(16) |
| *Scc9* | D10M46 | 10 | 63 | CcS-3,5 | D10M233(62)-Telomere(77) |
| *Scc11* | D4M11 | 4 | 57.4 | CcS-19 | D4M37(56.5)-D4M70(62.3) |
| *Scc12* | D7M67 | 7 | 63.5 | CcS-19 | D7M164(60)-Telomere(72.4) |
| *Scc13* | D6M122 | 6 | 29 | CcS-19 | D6M73(28)-D6M320(32.5) |
| *Scc14* | D10M75 | 10 | 2 | CcS-19 | Acromere(0)-D10M3(21) |
| *Scc15* | D11M26 | 11 | 33.9 | CcS-19 | D11M112(32)-D11Nds18(40) |

Detailed information of all the *Sluc* and *Scc* loci mapped previously are listed, including the microsatellite markers with linkage, their chromosomes and positions in cM, the strains in which they were detected, and their maximal candidate regions.
