# Supplementary figures and images for: Most Lung and Colon Cancer Susceptibility Genes Are Pair-Wise Linked in Mice, Humans and Rats
Source: PLoS One. 2011 Feb 24;6(2):e14727. doi: 10.1371/journal.pone.0014727 (PMC3044722; doi:10.1371/journal.pone.0014727)

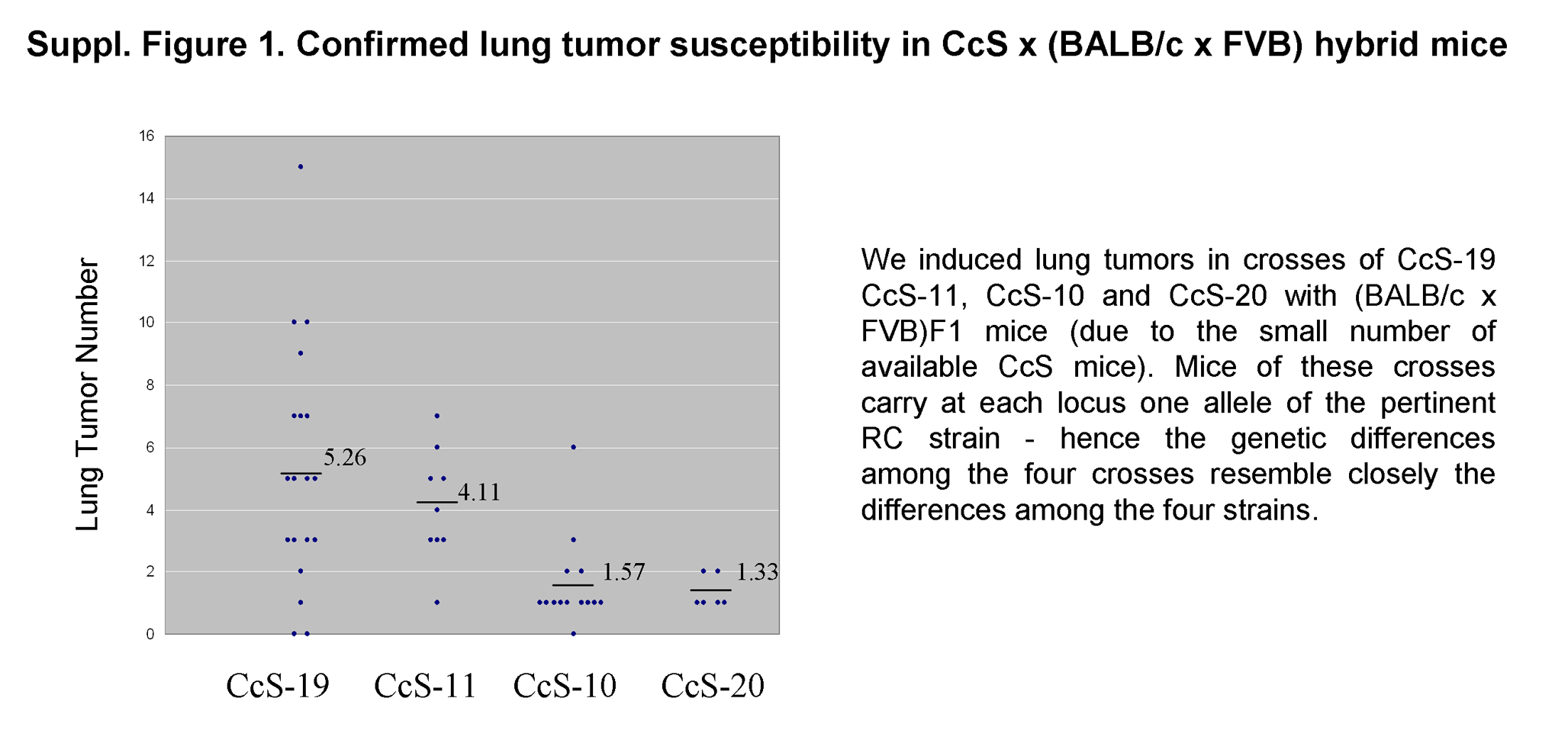

Supplement: Figure S1 — Supplementary Figure 1. (0.28 MB TIF) [file pone.0014727.s004.tif]
